# Ventilator-Associated Lower Respiratory Tract Infections and Their Association With COVID-19: A Retrospective Cohort Study in a Portuguese Intensive Treatment Unit

**DOI:** 10.7759/cureus.54108

**Published:** 2024-02-13

**Authors:** André Fernandes, Joao Nuno Patricio, Rita Jorge, Raquel Nazareth, Carlos S Pereira

**Affiliations:** 1 Internal Medicine, Hospital Beatriz Ângelo, Loures, PRT; 2 Intensive Care Unit, Hospital Beatriz Ângelo, Loures, PRT

**Keywords:** critical care, antimicrobial resistance, nosocomial infection, ventilator-associated pneumonia, invasive mechanical ventilation, covid-19

## Abstract

Introduction

Ventilator-associated pneumonia (VAP) is the most common infectious complication related to admission to an Intensive Treatment Unit (ITU). Ventilator-associated lower respiratory tract infection (VA-LRTI) is a broader diagnosis than VAP. By disregarding radiological criteria, it will include both VAP and ventilator-associated tracheobronchitis. This study, conducted in the setting of a Portuguese ITU, aims to study the incidence, microbiology and clinical outcome of VA-LRTI and its association with COVID-19.

Methods

A retrospective cohort study included patients admitted to a Portuguese ITU who underwent invasive mechanical ventilation (IMV) for over 48 hours between 01/01/2021 and 31/12/2021. The Hospitals in Europe Link for Infection Control through Surveillance (HELICS) criteria were applied, disregarding the radiological criteria, for the diagnosis of VA-LRTI.

Results

The group of patients with COVID-19 had 46.38 episodes of VA-LRTI/1000 days of ventilation, while patients without COVID-19 had 16.35 episodes/1000 days of ventilation (RR 2.78, p < 0.001). Of the 85 microorganisms isolated, 82% were gram-negative microorganisms, with species of the genus *Klebsiella* being the most prevalent (22.4%). There was a lower prevalence of beta-lactam-resistant organisms in patients with COVID-19 (RR 0.35, p = 0.031). The development of VA-LRTI is associated with longer times of IMV (difference in medians 10 days, p < 0.001), but with no significant differences in mortality (RR 1.21, p = 0.14).

Discussion

Patients with COVID-19 seem more predisposed to developing VA-LRTI, possibly due to intrinsic characteristics of the disease. Although no increase in mortality has been demonstrated, VA-LRTI can entail important costs related to morbidity, antibiotic pressure and economic costs that must be considered.

## Introduction

Ventilator-associated pneumonia (VAP) is the most common infectious complication associated with intensive treatment unit (ITU) stay [1]. The development of VAP is associated with considerable morbidity, including increased ITU length of stay, increased invasive mechanical ventilation (IMV) time and increased hospitalization costs [2].

VAP is defined as bacterial pneumonia that develops after 48 hours of IMV, requiring, according to the Hospitals in Europe Link for Infection Control through Surveillance (HELICS) definition, radiological, clinical and microbiological criteria to make the diagnosis [3]. The usage of a radiological criterion, however, may pose some serious difficulties, due to the interindividual variability of the evaluation and increased difficulties interpreting de novo pulmonary infiltrates in the complex ITU setting. This problem is of particular relevance in patients with viral pneumonia, such as Coronavirus Disease 2019 (COVID-19), whose lung parenchyma may be already severely affected by the underlying disease. For this reason, several authors have been working under the concept of ventilator-associated lower respiratory tract infections (VA-LRTI), which includes both VAP and ventilator-associated tracheobronchitis (the latter being distinguished from the first by the absence of pulmonary infiltrates), disregarding the radiological criterion [4-7].

Between 2020 and 2023, the world faced the emergence of the COVID-19 pandemic, arising a new population of patients admitted to hospital environment worldwide, namely ITU, and requiring IMV. This population of patients represented a new challenge regarding ventilatory support and antimicrobial strategies. Even so, as far as the knowledge of the authors goes, there are no published studies in Portugal regarding the impact of this disease in the development of VA-LRTI and its outcome. The aim of this work is to study the incidence, microbiology and clinical outcome of VA-LRTI and their association with COVID-19 in a Portuguese ITU.

## Materials and methods

This work is a retrospective cohort study conducted in an ITU of a Portuguese Group I Hospital (primarily serving area of less than 500,000 people and no secondary serving population). The inclusion criteria included all adult (age above 18) patients admitted to the ITU between 01/01/2021 and 31/12/2021 who underwent IMV, regardless of the cause. Patients ventilated for less than 48 hours were excluded. For data collection, the existing databases in the ITU were used, which contained demographic information, admission length, clinical outcomes and medical complications.

HELICS criteria were used, except for the radiological criterion, for establishing the diagnosis of VA-LRTI. Classification according to microbiological isolates can be found in Table 1. Bronchoalveolar lavage and tracheal aspirate specimens were incubated in no selective media, Haemophilus selective media (Columbia agar) and MacConkey media for 48 hours. Blood cultures were processed in BACT/ALERT® (bioMérieux, Marcy-l'Étoile, France) system for five days and incubated in Columbia agar and Schaedler agar if positive. Antimicrobial susceptibility tests were performed using phenotypic methods. SARS-CoV-2 positivity was determined through RT-PCR in any respiratory sample. Considering the retrospective nature of the study, clinical criteria were applied according to the responsible medical team judgment. When mortality rate is referred, intra-hospital mortality under current admission is considered, and not only the ITU mortality rate. VA-LRTI that developed in less than five days of IMV was classified as early VA-LRTI, whilst VA-LRTI occurring ≥5 days of IMV was classified as late VA-LRTI.

**Table 1:** HELICS classification for VAP, according to microbiological criteria. HELICS - Hospitals in Europe Link for Infection Control through Surveillance, PN - Pneumonia, VAP - Ventilator-Associated Pneumonia

Subgroup	Diagnostic method
PN1	Bacteriological diagnosis based on quantitative analysis of a minimally contaminated sample from the lower respiratory tract (e.g., bronchoalveolar lavage)
PN2	Bacteriological diagnosis based on quantitative analysis of possibly contaminated sample from the lower respiratory tract (e.g., tracheal aspirate)
PN3	Alternative microbiological diagnosis (e.g., blood cultures, pleural fluid)
PN4	Sputum culture or non-quantitative analysis of lower airway sample
PN5	No positive microbiological results.

Statistical analysis was performed using IBM SPSS Statistics 28.0 (IBM Corp., Armonk, NY, USA). Continuous variables were described using mean and standard deviation (SD) or median and interquartile range (IQR) and categorical variables through absolute value and proportions. Mann-Whitney U tests were performed to determine the statistical significance of continuous variables and Chi-Square (χ^2^) tests for categorical variables. For the VA-LRTI incidence calculation and its group effects, Poisson regression was used. Furthermore, Kaplan-Meier curves were plotted regarding the probability of development of VA-LRTI against mechanical ventilation length. All statistical tests performed were two-tailed, with statistical significance reached at a p < 0.05 level.

## Results

Population characteristics and VA-LRTI incidence

The study included 274 patients who were submitted to IMV for more than 48 hours, 139 (50.7%) of which had the diagnosis of COVID-19. Of the 135 (49.3%) without COVID-19, 89 (65.9%) had a medical cause for admission, and 46 (34.1%) had a surgical cause.

In Table 2, the characteristics of both groups are summarized. COVID-19 patients were younger (average age 60.8 vs. 65 years), requiring longer times of IMV (median 9 vs. 5 days) and longer length of stay in the ITU (median 13 vs. 6.5 days). There were no statistically significant differences in the gender distribution between the two groups. The incidence of VA-LRTI was 46.38 episodes/1000 ventilator days in the group of patients with COVID-19 and 16.35 episodes/1000 ventilator days in the non-COVID-19 patients’ group, posing a statistically significant relative risk of 2.78. There was no statistically significant association between gender or age and the development of VA-LRTI.

**Table 2:** Characteristics of ITU stay and incidence of VA-LRTI in the COVID-19 and non-COVID-19 population. ep. - episodes, STD - standard deviation, IQR - interquartile range, IMV - Invasive Mechanical Ventilation, ITU - Intensive Treatment Unit, VA-LRTI - Ventilator-Associated Lower Respiratory Tract Infections

	COVID-19 (N = 139)	Non-COVID-19 (N = 135)		p
Age – mean (STD)	60.8 (12.19)	65.0 (14.1)	0.012	
Gender Female – n (%)	43 (31%)	52 (39%)	0.187	
Gender Male – n (%)	96 (69%)	83 (61%)	0.187	
Length of stay – median (IQR)	13 (8-19)	6.5 (4-13)	<0.001	
Length of IMV – median (IQR)	9 (5-16.5)	5 (3-10)	<0.001	
VA-LRTI episodes - n	79	19	-	
VA-LRTI incidence – (ep./1000 days of IMV)	46.38	16.35	<0.001	
Relative Incidence	2.78	1		
Days to VA-LRTI development after start of IMV – median (IQR)	5.0 (4-8)	6 (4-11)	0.786	

**Figure 1:**
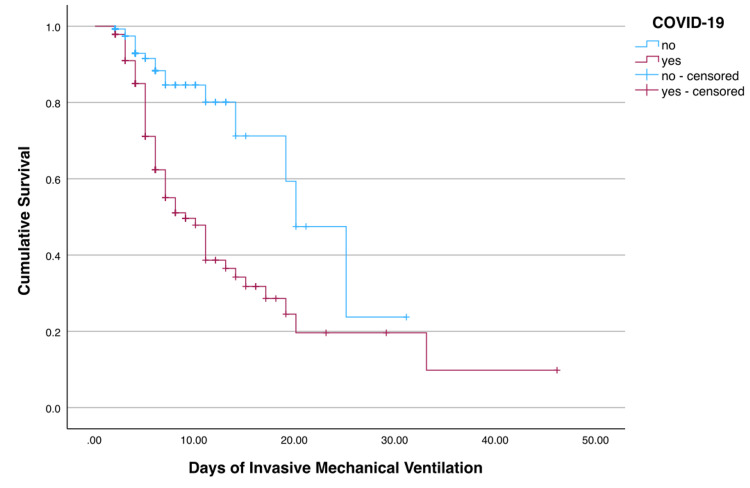
Kaplan-Meyer survival curve for the event "developing VA-LRTI". Censored events correspond to extubation (alive or dead). VA-LRTI - Ventilator-Associated Lower Respiratory Tract Infections

The median time to the development of the first VA-LRTI was 5 (IQR 4) days after initiation of mechanical ventilation and no statistically significant differences were found between both groups. In Figure 1, we plot the Kaplan-Meyer curves for the development of VA-LRTI. By this method, the median survival (free of VA-LRTI) is estimated at nine days for patients with COVID-19 and 20 days for patients without COVID-19, with a statistical significance of < 0.001 for the difference between the two groups.

In Table 3, we present the incidence of VA-LRTI by quarter. Of particular note is the predominance of COVID-19 episodes in the first trimester, but still without an increase of VA-LRTI in this period.

**Table 3:** Number of episodes (n) of IMV with ≥48 hours of ventilation and incidence of VA-LRTI, per quarter, in the groups of patients with and without COVID-19. ep. - episodes, IMV - Invasive Mechanical Ventilation, VA-LRTI - Ventilator-Associated Lower Respiratory Tract Infections

Time of Admission	COVID-19		Non-COVID-19	
	n	ep./1000 days of IMV	n	ep./1000 days of IMV
1^st^ Quarter	102	35.68	27	9.95
2^nd^ Quarter	13	60.98	41	7.27
3^rd^ Quarter	17	42.63	24	15.24
4^th^ Quarter	7	19.231	43	22.35

Type of VA-LRTI

**Figure 2:**
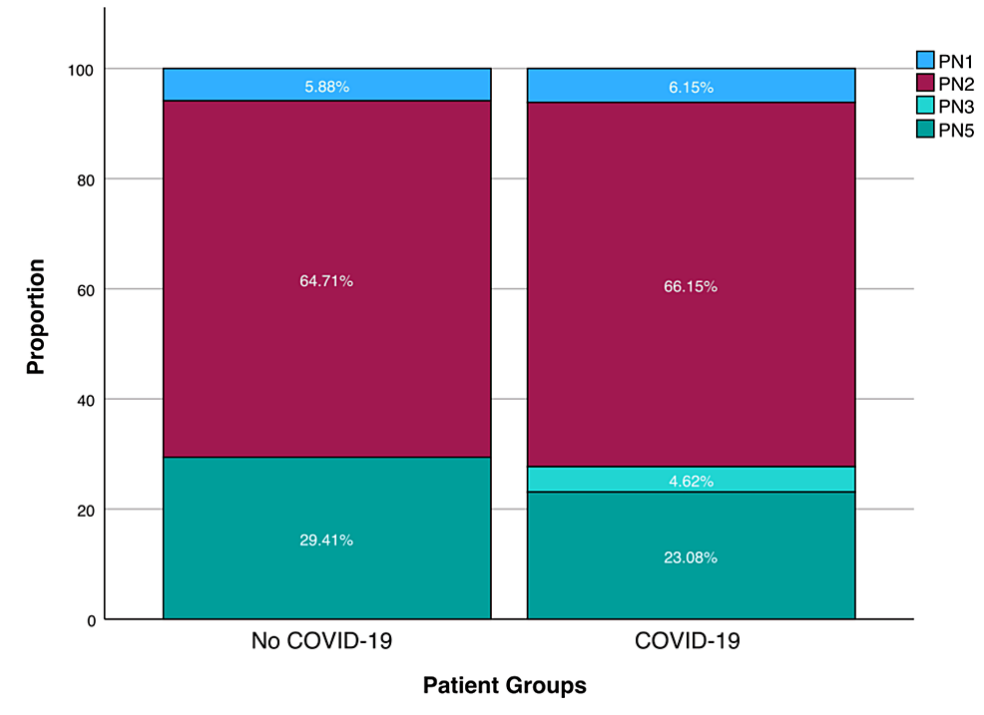
Relative frequency of VA-LRTI type by patient group (with and without COVID-19). VA-LRTI - Ventilator-Associated Lower Respiratory Tract Infections, PN - pneumonia (please refer to Table 1 for further information regarding subgroup classification)

The most frequent type of VA-LRTI was PN2, microbiological isolate in tracheal aspirate, summing up to 65.9% of recorded lower respiratory infections, followed by PN5, clinical diagnosis without microbiological criterion, which represents 24.4% of recorded lower respiratory infections. Figure 2 shows the distribution of VA-LRTI type by COVID-19 and non-COVID-19 groups. There is no statistically significant difference regarding the type of VA-LRTI between both groups (p = 0.798, χ^2^).

Microbiological isolates

There were 85 microorganisms isolated from a total of 66 patients. Amongst these microorganisms, the vast majority (82%) were gram-negative bacteria, with Klebsiella genus being the most prevalent (22.4%). Infections due to beta-lactam-resistant (BLR) bacteria - we included in this definition Methicillin-resistant *Staphylococcus aureus* (MRSA), broad-spectrum beta-lactam resistance (3rd generation cephalosporins and piperacillin/tazobactam) and carbapenem resistance - sum up to 21.2% of recorded infections, whilst 18.8% of isolates were resistant to amoxicillin (Table 4).

**Table 4:** Isolated bacteria (N = 85) in COVID-19 (N = 69) and Non-COVID-19 (N = 16) patients and their antimicrobial susceptibility profiles in VA-LTRI patients. ^a^ *S. pneumoniae* (n=3), *E. coli* (n=3), *Proteus spp.* (n=3), *Morganella spp.* (n=3), *Citrobacter spp.* (n=2), *Raoultella sp. *(n=2), *Stenotrophomonas sp. *(n=2), *Enterococcus sp. *(n=1), *Citrobacter sp. *(n=1), *Moraxella sp. *(n=1) ^b^ Intrinsic resistances were not considered

Bacteria	TOTAL n (%)	COVID-19 n (%)	Non-COVID-19 n (%)	p-value
Klebsiella spp.	19 (22.4%)	14 (20.3%)	5 (31.3%)	0.343
Pseudomonas aeruginosa	13 (15.3%)	7 (10.1%)	6 (37.5%)	0.006
Staphylococcus aureus	11 (12.9%)	8 (11.6%)	3 (18.8%)	0.442
Enterobacter spp.	9 (10.6%)	9 (13.0%)	0 (0%)	0.127
Haemophilus spp.	6 (7.1%)	6 (8.7%)	0 (0%)	0.221
Serratia sp.	6 (7.1%)	5 (7.2%)	1 (6.3%)	0.889
Other ^a^	21 (24.7%)	20 (29%)	1 (6.3%)	0.057
Antimicrobial Resistance ^b^				
Bacteria with no acquired resistance	51 (60%)	44 (63.8%)	7 (43.8%)	0.141
Amoxicillin-resistant gram-negatives	16 (18.8%)	15 (21.7%)	1 (6.3%)	0.153
Methicillin-resistant Staphylococcus aureus	3 (3.5%)	2 (12.5%)	1 (1.4%)	0.031
Gram-negatives resistant to broad-spectrum beta-lactams	10 (11.8%)	7 (10.1%)	3 (18.8%)	0.336
Gram-negatives resistant to carbapenems	5 (5.9%)	2 (2.9%)	3 (18.8%)	0.015

We did not find any statistically significant correlation between the timing of development of VA-LRTI (early vs. late) and the prevalence of BLR bacteria. On the other hand, there is a strong association between the prevalence of BLR bacteria and previous exposure to antimicrobials (p = 0.005), with nil BLR bacteria isolated in patients who were not yet exposed to antimicrobials. With that regard, we considered previous antimicrobial exposition to any antimicrobial treatment during the admission episode under analysis, including before orotracheal intubation. Furthermore, the prevalence of BLR bacteria in COVID-19 patients appears to be lower (Table 5).

**Table 5:** Prevalence of BLR microorganisms according to the group, timing of presentation and previous antimicrobial exposure. BLR - Beta-lactam-resistant, VA-LRTI - Ventilator-Associated Lower Respiratory Tract Infections.

Group	BLR bacteria (%)	p
COVID-19	16.0%	0.031
Non-COVID-19	45.5%	
Early VA-LRTI	16.7%	0.567
Late VA-LRTI	23.3%	
Previous antimicrobial exposition	0%	0.005
No previous antimicrobial exposition	31.7%	

Clinical outcome - mortality and length of invasive mechanical ventilation

There is a statistically significant association between the development of VA-LRTI and the average length of IMV, both in patients with COVID-19 and without COVID-19 (difference in medians 10 and 10.5, respectively, p-values <0.001). On the other hand, the superinfection with a BLR bacteria is not significantly related to a longer length of mechanical ventilation (p = 0.312).

Regarding mortality, our results could not establish an increased risk of intra-hospital mortality of patients with VA-LRTI when compared with patients without VA-LRTI. The superinfection with a BLR bacteria, on the contrary, is related to a higher intra-hospital mortality rate (mortality rate 84.6%, RR 1.69, p = 0.025) (Table 6).

**Table 6:** Length of IMV and mortality in patients with and without development of VA-LRTI and subgroup analysis. IMV - Invasive mechanical ventilation; VA-LRTI - Ventilator-Associated - Lower Respiratory Tract Infection; IQR - Interquartile range; BLR - Beta-lactam-resistant

a) Days of IMV				
	VA-LRTI median (IQR)	Non-VA-LRTI median (IQR)	Difference in Medians	p-value
Global	15 (10-24)	5 (3-8.5)	10	<0.001
COVID-19	15 (10-23)	5 (3-9)	10	<0.001
Non-COVID-19	15 (11-36)	4.5 (3-8)	10.5	<0.001
Non-BLR bacteria	15 (9-23)	-	-	0.312
BLR bacteria	17 (14-27)	-	-	
b) Mortality				
	VA-LRTI % (n/N)	Non-VA-LRTI % (n/N)	Relative Risk (VA-LRTI/Non-VA-LRTI)	p-value
Global	56.1% (46/82)	46.4% (89/192)	1.21	0.14
COVID-19	52.3% (34/65)	41.9% (31/74)	1.25	0.219
Non-COVID-19	70.6% (12/17)	49.2% (58/118)	1.43	0.098
Non-BLR bacteria	50% (24/48)	-	-	0.025
BLR bacteria	84.6% (11/13)	-	-	

## Discussion

According to our results, COVID-19 patients have a higher incidence of VA-LRTI when compared with patients without COVID-19, with a relative risk of 2.76 (p <0.001). This is in line with other studies available, which have already demonstrated an increased risk of VAP or VA-LRTI in this population. The reported incidence of VAP/VA-LRTI in COVID-19 patients spans between 18-39 ep./1000 days of ventilation [5,8-10], which is less than in our population. We should, however, emphasize that three out of four of the aforementioned studies [5,8,10] only included episodes with microbiological confirmation of a VA-LRTI, which may underestimate the real number of infections.

One might be tempted to argue that there might be an overdiagnosis (when only clinical criteria are used) of VA-LRTI in this population due to the increased difficulties in diagnosing a pulmonary superinfection in a patient whose lung parenchyma is already extensively damaged by the underlying viral infection. However, the similar distribution of type of VA-LRTI between the two groups won’t support this hypothesis. In fact, in most patients with COVID-19, a microbiological isolate was identified in a respiratory sample. On the other hand, it is also unlikely that comorbidities will be responsible for the higher VA-LRTI incidence (even though the model wasn’t adjusted for them), given patients with COVID-19 were younger than the counterpart.

As for the reasons why that happens, we can hypothesize that it is due to factors intrinsic to the disease itself, or extrinsic factors, such as the number of prone positionings or the high use of corticosteroids. Two recent studies have established a relationship between dexamethasone and the incidence of VAP, although with no increase in mortality [11,12]. Regarding prone positioning, on the other hand, most of the research works have tried to demonstrate a protective effect of prone position in the development of VAP, although no statistical significance is usually achieved [13,14]. Furthermore, an investigation work during a SARS-CoV outbreak, in a Hong Kong hospital in 2004, reported a VAP incidence of 36.5 ep./1000 days of mechanical ventilation, which supports the hypothesis of a characteristic intrinsic to the disease [15].

It should also be noted that these data refer to the year 2021, spanning approximately from the third wave (when most stress was put upon the Portuguese health system) to the beginning of the fifth wave of the COVID-19 pandemic. Even so, the incidence of VA-LRTI doesn’t seem to have increased in the first quarter of that year, which suggests that primary preventive measures (as in the “VAP bundle”) were still attained regardless of the pressure on the system. Data regarding VA-LRTI in the following quarter should be interpreted with increased caution, due to the low number of patients. A comparative analysis against the first wave would also be interesting, considering corticosteroid use during that period was considerably different. Adenosine analogues or interleukin-6 inhibitors were not routinely used in our ITU, and thus its potential impact on the results can be disregarded.

We found differences not only in the incidence rates, but also in some characteristics of the microorganisms isolated in COVID-19 patients when compared to the non-COVID-19 counterpart. For instance, there is a significantly decreased rate of Pseudomonas aeruginosa (RR 0.27, p = 0.006) and a significantly decreased rate of BLR bacteria (RR 0.35, p = 0.032) in COVID-19 patients. That is probably explained by the fact that COVID-19 brought to the ITUs an unprecedented number of patients with no previous diseases or attendance to health institutions.

Previous exposition to antimicrobials was proved to be a good predictor of a BLR bacteria isolate, whilst the timing of development of VA-LRTI didn’t. That is likely related to the fact that even the patients who develop a VA-LRTI early in their ventilation course may have been exposed to BLR bacteria earlier in their hospital admission (or previous admissions), prior to orotracheal intubation. The superinfection with a BLR bacteria results in a higher risk of death (RR 1.69, p = 0.025), but no impact on the length of mechanical ventilation (p = 0.932).

Still regarding the microorganisms, a last note is given to the low prevalence of MRSA infections (3.5% of isolates), which suggests that vancomycin should be used judiciously in our population.

Despite a clear association between VA-LRTI and the length of mechanical ventilation, our results couldn’t prove a statistically significant relation between VA-LRTI and intra-hospital mortality. This tendency, however, is stronger in the population of patients without COVID-19. These results are in accordance with another study, where the development of VAP was a predictor of death in patients without COVID-19, but not in COVID-19 patients [16]. Nonetheless, even without a proven increase in mortality, VA-LRTI may withstand significant costs related to morbidity, antimicrobial pressure and economic costs that were not addressed in this study.

The main limitations of this research are its retrospective nature, difficulties related to access of additional data from clinical records and the fact that it was done in a single center, which may limit the external extrapolation of its results. Morbidity data, such as the performance status at discharge or morbimortality at 6 and 12 months, which might have given an important insight into the VA-LRTI burden, were also not analyzed.

## Conclusions

COVID-19 patients have a higher incidence of VA-LRTI when compared with patients without COVID-19, probably due to characteristics intrinsic to the disease. There is a significantly decreased rate of BLR bacteria (RR 0.35, p = 0.032) in COVID-19 patients. Previous exposition to antimicrobials was proved to be a good predictor of a BLR bacteria isolate, whilst the timing of development of VA-LRTI didn’t. Despite a clear association between VA-LRTI and the length of mechanical ventilation, our results couldn’t prove a statistically significant relation between VA-LRTI and intra-hospital mortality.

This research demonstrates some aspects of uttermost importance to the knowledge of SARS-CoV-2 disease, particularly relevant at a local and national level because it is the only one conducted in the Portuguese population, and may support further investigations.
